# Thermodynamics of Composition Graded Thermoelastic Solids

**DOI:** 10.3390/e25071084

**Published:** 2023-07-19

**Authors:** Vito Antonio Cimmelli

**Affiliations:** Department of Mathematics, Computer Science and Economics, University of Basilicata, Viale dell’Ateneo Lucano, 10, 85100 Potenza, Italy; vito.cimmelli@unibas.it

**Keywords:** composition graded materials, thermoelastic solids, generalized Coleman–Noll procedure, thermoelastic-wave propagation

## Abstract

We propose a thermodynamic model describing the thermoelastic behavior of composition graded materials. The compatibility of the model with the second law of thermodynamics is explored by applying a generalized Coleman–Noll procedure. For the material at hand, the specific entropy and the stress tensor may depend on the gradient of the unknown fields, resulting in a very general theory. We calculate the speeds of coupled first- and second-sound pulses, propagating either trough nonequilibrium or equilibrium states. We characterize several different types of perturbations depending on the value of the material coefficients. Under the assumption that the deformation of the body can produce changes in its stoichiometry, altering locally the material composition, the possibility of propagation of pure stoichiometric waves is pointed out. Thermoelastic perturbations generated by the coupling of stoichiometric and thermal effects are analyzed as well.

## 1. Introduction

Functionally graded systems are new materials whose behavior varies along some given directions inside the system [1,2]. Such a property can be used to optimize their performances in several applications [3]. For example, at nanoscale, graded alloys of type ${\mathrm{Si}}_{c}{\mathrm{Ge}}_{1-c}$ with the stoichimetric variable $c\in \left[0;1\right]$ changing along a given direction are used in the design of thermal diodes [4,5,6,7,8,9,10] or to enhance the efficiency of thermoelectric energy conversion [11,12]. Graduation can be also induced by the anisotropic dependency of the properties of the material on spatial gradients of some thermodynamic variables, since a spatial gradient in the microstructure along a certain direction results in changes in the local material properties. For instance, many biological materials exhibit spatial gradients in the local chemical composition, which can be used to improve the mechanical properties of biomaterials and to activate their functionality. In the last decades, substantial effort was dedicated to fabricating functionally graded materials, with the scope of controlling their physical properties such as heat conduction, electric conductivity and resistance to enhanced stresses. That research ranged over a characteristic length scale from nanometers to millimeters [13]. The study of elastic and thermal properties of composition graded systems is a promising field of research, since applied mechanical stresses may influence their thermal conductivity, thus providing a wider degree of control of their thermal behavior [8,14,15].

In the present paper, we focus on the thermodynamics of composition graded thermoelastic systems, by postulating a set of balance equations modeling their behavior and investigating its compatibility with second law of thermodynamics [16]. A system of thermodynamic restrictions, giving necessary and sufficient conditions for the thermodynamic compatibility, is derived by applying a generalized Coleman-Noll procedure for the exploitation of the entropy inequality [17,18,19]. Furthermore, we investigate the propagation of thermoelastic waves. We derive the necessary and sufficient conditions for the propagation of the acceleration waves and analyze them in different physical situations. In such a way, we obtain useful information on what could be derived from experimental thermoelastic–perturbation measurements and how such measurements can be used in material design and fabrication process.

Our investigation is focused on graded systems at a nanometric scale. In nanosystems, nonlinear effects influence the propagation of thermomechanical disturbances [20,21,22,23]. Then, new mathematical models beyond the classical thermoelasticity [20,24,25,26,27,28] are necessary. In fact, at nanoscale, even small differences in temperature or displacement may produce strong gradients in such a way that linear constitutive equations are no longer valid. Furthermore, when a transient thermal pulse is applied to one end of a system, it arrives at the opposite end in a very short time, i.e., before reaching its equilibrium value. Thus, when studying thermoelastic nanosystems, a nonlinear heat-transport equation allowing propagation of thermomechanical disturbances with a finite speed is also needed. Due to the considerations above, we assume that:

- the heat-transport equation couples thermal and elastic effects;
- the effective thermal conductivity and the relaxation time depend on deformation, too;
- the stoichiometric variable c∈[0,1] and its gradient enter the state space;
- the variable *c* can change with time due to the changes in volume produced by deformation and thermal expansion.

The meaning of the last condition is that, in practice, we regard *c* as a a scalar internal variable whose dependency on the points of the system is known when the process starts, but changes as a consequence of thermoelastic deformation. As usual for internal variables, the kinetic equation giving the evolution of *c* is defined on the state space. It is worth observing the derivation of such equation does not follow either from experimental results or from kinetic modeling because both are lacking in the literature, but it is a theoretical postulate based on the assumption that changes in strain and temperature necessarily induce variation in the composition. In order to justify such an assumption, one should note that variable composition is related to the internal microstructure, such as, for instance, the presence of a grain structure, of dislocations, or of multiple metastable phases, leading to multiple deformation mechanisms which can be manipulated by controlling the local chemistry [29]. Then, to our view, time-dependent deformation and heating of such material necessarily lead to changes in time of microstructure, i.e., to a time dependency of the stoichiometric variable. We observe that in future research such a hypothesis can be tested by comparing the results on the speeds of thermomechanical disturbances shown in Section 4 with numerical simulation of wave propagation, (see, for instance, Ref. [30]). The usefulness of the present analysis becomes more evident if one considers that the variation with respect to time of *c* can be established in the phase of fabrication of the graded material in order to optimize its performance. In the following, we focus on small deformations so that our model equations remain within the frame of linear thermoelasticity, although due to the nonlinear dependency of the constitutive equations on the strain and on the heat flux, the full model is nonlinear. In such a framework we develop a mathematical model which couples two thermodynamic theories, namely Rational Thermodynamics [17,31] and Extended Irreversible Thermodynamics [25]. The following set of equations is postulated.

(1a) $$\begin{array}{cc} \; & \varrho {\ddot{u}}_{i}-T_{ij,j}=0, \end{array}$$

(1b) $$\begin{array}{cc} \; & \varrho \dot{\varepsilon }-T_{ij}{\dot{E}}_{ij}+q_{i,i}=0, \end{array}$$

(1c) $$\begin{array}{cc} \; & \tau_{q}A_{q}{\dot{q}}_{i}+q_{i}+\bar{\kappa }A_{K}\varepsilon_{,i}-\frac{2\tau_{Q}}{\varrho \varepsilon }q_{j,i}q_{j}-\tau_{E}{\dot{E}}_{ij}q_{j}-a_{c}c_{,i}=0, \end{array}$$

(1d) $$\begin{array}{cc} \; & \dot{c}=H\left(\Sigma \right), \end{array}$$

where

- the indices i,j, range from 1 to 3, and notation $f_{,i}$ means the partial derivative of the regular function *f* with respect to the cartesian coordinates $x_{i}$;
- $u_{i}$ is the local displacement vector with respect to a reference material configuration;
- $T_{ij}$ is the (symmetric) Cauchy stress tensor;
- ϱ is the mass density;
- ε is the internal energy per unit mass;
- $q_{i}$ is the local heat flux;
- $E_{ij}$ is the strain tensor (i.e., $E_{ij}=\frac{u_{i,j}+u_{j,i}}{2}$);
- $\bar{c}$ is the specific heat related to the internal energy per unit of mass by relation $\varepsilon =\bar{c}\vartheta$, where $\vartheta \equiv {\left(\frac{\partial s}{\partial \varepsilon }\right)}^{-1}$ is the absolute temperature;
- κ is the thermal conductivity and $\kappa A_{K}\equiv \kappa \left(1+a_{\kappa }E_{hk}E_{hk}\right)$ is the effective thermal conductivity, depending on the deformation;
- $\bar{\kappa }=\kappa /\bar{c}$;
- $\tau_{q}$ is a material parameter, depending on ε and *c*, such that $\tau_{q}A_{q}\equiv \tau_{q}\left(1+a_{\tau }E_{hk}E_{hk}\right)$ represents the total relaxation time of the heat flux (i.e., the time elapsed between the application of a difference of temperature and the appearance of a heat flux);
- $\tau_{E}$ is the relaxation time of the strain (i.e., the time elapsed between the application of a stress and the appearance of a deformation);
- $\tau_{Q}$ is the relaxation time of the heat carriers, i.e., the quantity $\frac{\ell }{\bar{v}}$, with *ℓ* as the mean free path and $\bar{v}$ as the mean speed of the heat carriers (phonons, electrons, holes);
- $a_{\tau }$, $a_{\kappa }$ and $a_{c}$ are material functions depending on ε and *c*;
- Σ denotes the set of the elements of the state space which is specified better below.

Equations (1a)–(1d) represent an undetermined system of 8 partial differential in the 15 unknown quantities $u_{i},\;T_{ij},\;q_{i},\;\varepsilon ,\;c,\;H$. Then, seven additional constitutive equations for $T_{ij}$ and *H* are needed. Those equations are defined on the following state space
  (2) $$\Sigma \equiv \left\{\varepsilon ,\varepsilon_{,k},c,c_{,k},q_{h},q_{h,k},E_{hk}\right\}.$$
The second law of thermodynamics imposes that the local rate of entropy production
  (3) $$\sigma^{s}\equiv \rho \dot{s}+J_{i,i}\;,$$
  with *s* as the internal entropy per unit of mass and $J_{i}$ as the local entropy flux, has to be nonnegative for arbitrary thermodynamic processes [16], i.e.,
  (4) $$\rho \dot{s}+J_{i,i}\geq 0\;.$$
The dissipation principle, postulated by Coleman and Noll in 1963 [17,32,33], requires that the constitutive equations for the seven constitutive quantities $T_{ij}$ and *H* must be assigned in such a way that the unilateral differential constraint (4) is satisfied whatever the solution of the system (1a)–(1d) is.

We note that in Equation (1c), the strain influences both the total relaxation time and the heat conductivity according to the results in [34,35]. We also note that we are supposing $\tau_{q}\neq \tau_{Q}$. Indeed, at nanoscale size, effects on the relaxation of the heat flux become as mportant as the characteristic dimension decreases. Consequently, in the present approach, the total relaxation time of the heat flux (namely $\tau_{q}$) does not coincide with the relaxation time of the heat carriers (namely $\tau_{Q}$) [36,37]. At macroscopic scale, instead, where the size effects are absent, $\tau_{q}$ reduces to $\tau_{Q}$, and the strain does not influence the evolution equation of the heat flux, so that $\tau_{E}=a_{\tau }=a_{\kappa }=0$. Then, Equation (1c) reduces to

(5) $$\tau_{Q}{\dot{q}}_{i}+q_{i}+\frac{\kappa }{\bar{c}}\varepsilon_{,i}-\frac{2\tau_{Q}}{\varrho \varepsilon }q_{j,i}q_{j}=0,$$

which is the nonlinear Maxwell–Cattaneo equation derived in [38,39].

The novelties of the present research can be summarized as follows:

- a thermodynamic model of composition graded material with nonlocal constitutive equations is studied, for the first time, by a generalized Coleman–Noll procedure;
- the specific entropy and the stress tensor of the material at hand may depend on the gradient of the unknown fields;
- several different types of coupled first- and second-sound pulses, depending on the value of the material coefficients characterizing the system, may propagate;
- for the first time, the possibility of propagation of pure stoichiometric waves generated by the presence of a composition gradient is proved.

The paper has the following layout.

In Section 2, we derive by some general physical laws the model equations postulated in Section 1.

In Section 3, we prove the compatibility of such equations with the second law of thermodynamics and derive some possible expressions of the Cauchy stress tensor, of the specific entropy, and of the time derivative of *c* (function *H*).

In Section 4, we calculate the speeds of propagation of thermoelastic pulses both in equilibrium states and in nonequilibrium states.

In Section 5, a discussion of the obtained results is given, and possible developments of the theory are analyzed.

## 2. Balance Equations and Entropy Inequality

In this section, we point out the fundamental physical laws on which Equations (1a)–(1d) are based and derive those equations by such laws. We let B be a continuous body and b⊂B a compact and connected subset of B. We call b the material particle included in B. The scalars,

(6) $$Q_{i}\left(c,t\right)=\int_{b}\varrho {\dot{x}}_{i}db\;,$$

with ${\dot{x}}_{i}$ as the components of the velocity of the points of b, denote the components of the linear momentum of b. Then, the balance of linear momentum of b can be written as

(7) $$\frac{d}{dt}Q_{i}\left(b,t\right)=F_{i}\left(b,t\right)\;,$$

wherein $F_{i}\left(b,t\right)$ are the components of the total force acting on b. It is assumed that [17]

(8) $$F_{i}\left(b,t\right)=\int_{b}\rho b_{i}db+\int_{\partial b}t_{i}d\sigma \;,$$

wherein $b_{i}$ are the components of the body force acting on the points of B, and $t_{i}$ are the components of the surface force acting on ∂b due to the interaction of b with the external world.

**Theorem** **1.** *If $b_{i}=0$, Equations (7) and (8) hold for an arbitrary material particle b if, and only if, Equation (1a) is true.*

**Proof.**  The celebrated Cauchy theorem on the stress allows to write

(9) $$\int_{\partial b}t_{i}d\sigma =\int_{\partial b}T_{ij}n_{j}d\sigma =\int_{b}T_{ij,j}db\;,$$

wherein $n_{j}$ are the components of the exterior normal to ∂b and $T_{ij}\left(x,t\right)$ are the components of the Cauchy stress tensor. By the balance of angular momentum, it follows that the stress tensor is symmetric [17]. Thus, Equation (7) writes as

(10) $$\frac{d}{dt}\int_{b}\rho {\dot{x}}_{i}db=\int_{b}\rho b_{i}db+\int_{b}T_{ij,j}db\;.$$

By applying the transport theorem to the left-hand side of Equation (10), we finally obtain

(11) $$\int_{b}\left[\rho {\ddot{x}}_{i}-\rho b_{i}-T_{ij,j}\right]db=0\;,$$

with the superposed dot denoting the material time derivative. Due to the arbitrariness of the domain b and to the regularity of the integrand function, Equation (11) holds if, and only if,

(12) $$\rho {\ddot{x}}_{i}-\rho b_{i}-T_{ij,j}=0\;,$$

which represents the local balance of linear momentum. In the absence of body forces ($b_{i}=0)$, once the displacement vector $u_{i}=x_{i}-X_{I}$, with $X_{I}$ as the coordinates of the points of B in the reference configuration, Equation (12) reduces to Equation (1a). □

The first law of thermodynamics in a differential form reads

(13) $$\frac{dU}{dt}=\frac{dQ}{dt}-\frac{dL}{dt}\;,$$

wherein *U* is the total internal energy of the system, *L* is the work performed by the system toward the external ambient, and *Q* is the heat exchanged by the system along the thermodynamic transformation. On the other hand, we can suppose that

(14) $$\frac{dL^{e}}{dt}=\frac{dE_{k}}{dt}-\frac{dL}{dt}\;,$$

wherein $E_{k}$ is the total kinetic energy of the system, and $L^{e}$ is the work performed by the external forces on the system. Therefore, we obtain

(15) $$\frac{dU}{dt}=\frac{dQ}{dt}+\frac{dL^{e}}{dt}-\frac{dE_{k}}{dt}\;.$$

**Theorem** **2.** *In the absence of external heat souce, Equation (15) holds for an arbitrary material particle b if, and only if, Equation (1b) is true.*

**Proof.**  We first suppose that there exists a regular function ε, the internal energy per unit of mass, such that

(16) $$U=\int_{b}\rho \varepsilon db\;.$$

Thus,

(17) $$\frac{dU}{dt}=\frac{d}{dt}\int_{b}\rho \varepsilon db=\int_{b}\rho \dot{\varepsilon }db\;.$$

Moreover, along with Coleman and Noll [17], it is assumed that

(18) $$\frac{dQ}{dt}=\int_{b}\rho rdb-\int_{\partial b}q_{i}n_{i}d\sigma =\int_{b}\rho rdb-\int_{b}q_{i,i}db\;,$$

with *r* as the radiative power per unit of mass absorbed by the system and $q_{i}$ as the components of the local heat flux on ∂b. Moreover,

$$\frac{dL^{e}}{dt}=\int_{b}\rho b_{i}{\dot{x}}_{i}db+\int_{\partial b}t_{i}{\dot{x}}_{i}d\sigma =\int_{b}\rho b_{i}{\dot{x}}_{i}db+\int_{\partial b}{\dot{x}}_{i}T_{ij}n_{j}d\sigma =\int_{b}\rho b_{i}{\dot{x}}_{i}db+\int_{b}{\left({\dot{x}}_{i}T_{ij}\right)}_{,j}db=$$

(19) $$=\int_{b}{\dot{x}}_{i}\rho b_{i}db+\int_{b}{\dot{x}}_{i,j}T_{ij}db+\int_{c}{\dot{x}}_{i}T_{ij,j}db\;.$$

On the other hand, the kinetic energy can be written as

(20) $$E_{k}=\int_{b}\frac{1}{2}\rho {\dot{x}}_{i}{\dot{x}}_{i}db\;.$$

Hence,

(21) $$\frac{d}{dt}E_{k}=\int_{b}\rho {\dot{x}}_{i}{\ddot{x}}_{i}db\;.$$

Finally, after substitution of the previous expressions in Equation (15), we obtain

(22) $$\int_{b}\rho \dot{\varepsilon }db=-\int_{b}q_{i,i}db+\int_{b}\rho rdb+\int_{b}{\dot{x}}_{i}\left(-\rho {\ddot{x}}_{i}+T_{ij,j}+\rho b_{i}\right)db+\int_{b}{\dot{x}}_{i,j}T_{ij}db\;.$$

Once the balance of linear momentum and the symmetry of $T_{ij}$ are taken into account, we obtain

(23) $$\int_{b}\left(\rho \dot{\varepsilon }+q_{i,i}-T_{ij}D_{ij}-\rho r\right)db=0\;,$$

wherein $D_{ij}=\frac{{\dot{x}}_{i,j}+{\dot{x}}_{j,i}}{2}$. Due to the arbitrariness of the domain b and to the regularity of the integrand function, Equation (23) holds if, and only if,

(24) $$\rho \dot{\varepsilon }+q_{i,i}-T_{ij}D_{ij}-\rho r=0\;.$$

Equation (24) is the local balance of the total energy, namely the first law of thermodynamics for the continuum at hand. When expressed in terms of displacement, it yields Equation (1b) in the absence of external heat source, i.e., if r=0. □

As far as the heat flux is concerned, we regard it as and independent thermodynamic variable entering the state space together with its gradient and endowed by its own governing equation. For such variable, we postulate a global balance equation for the rate of variation of the total heat flux in the form of

(25) $$\frac{d}{dt}\int_{b}\rho q_{i}db+\int_{b}\Phi_{ij,j}db=r_{i}+P_{i}\;,$$

wherein $\Phi_{ij}$ is the flux of heat flux, $r_{i}$ is the heat flux production not related to the time variation of the strain, and $P_{i}$ is the additional heat flux production generated by the strain rate. In other words, we are supposing that the velocity of deformation influences the evolution of the heat flux. Hence, we assume

(26) $$r_{i}=-\int_{b}\frac{\rho q_{i}}{\tau_{q}A_{q}}db\;,$$

and

(27) $$P_{i}=\int_{b}\frac{\rho \tau_{E}}{\tau_{q}A_{q}}{\dot{E}}_{ij}q_{j}db\;.$$

**Remark** **1.** *Although the inclusion of the heat flux in the state space copes with the tenets of Extended Irreversible Thermodynamics, assumption (27) goes beyond such a theory, because $P_{i}$ depends also on the rate of a thermodynamic variable. Thus, our system can be also regarded as a generalized rate-type material.*

**Theorem** **3.** *The global balance law (25), with $r_{i}$ and $P_{i}$ given by Equations (26) and (27), holds for an arbitrary material particle b if, and only if, Equation (1c) is true.*

**Proof.**  Once Equations (26) and (27) are taken into account, Equation (25) can be rearranged as

(28) $$\int_{b}\left[\rho {\dot{q}}_{i}+\Phi_{ij,j}+\frac{\rho q_{i}}{\tau_{q}A_{q}}-\frac{\rho \tau_{E}}{\tau_{q}A_{q}}{\dot{E}}_{ij}q_{j}\right]db=0\;.$$

Due to the arbitrariness of domain b and to the regularity of the integrand function, Equation (28) holds if, and only if,

(29) $$\rho {\dot{q}}_{i}+\Phi_{ij,j}+\frac{\rho q_{i}}{\tau_{q}A_{q}}-\frac{\rho \tau_{E}}{\tau_{q}A_{q}}{\dot{E}}_{ij}q_{j}=0\;.$$

Then, if the spatial variation of the material functions is negligible, Equation (1c) can be recovered under the constitutive assumption

(30) $$\Phi_{ij}=\left(\frac{\rho \bar{\kappa }A_{K}}{\tau_{q}A_{q}}\varepsilon -\frac{\tau_{Q}}{\varepsilon \tau_{q}A_{q}}q_{k}^{2}-\frac{\rho a_{c}}{\tau_{q}A_{q}}c\right)\delta_{ij}\;,$$

where $\delta_{ij}$ denotes the Kronecker symbol, i.e., the identity matrix. □

For composition variable *c*, the general balance equation,

(31) $$\frac{d}{dt}\int_{b}\rho cdb+\int_{\partial b}C_{i}n_{i}d\sigma =\int_{b}\rho Hdb\;,$$

with $C_{i}$ as the flux of *c* through ∂b and *H* as the production of *c* per unit of mass, can be postulated. However, here, we regard *c* as an internal variable whose evolution is related to the variation of the internal composition of B. As a consequence, the flux of *c* through the boundary is absent, i.e., $C_{i}=0$.

**Theorem** **4.** *If $C_{i}=0$, Equation (31) holds for an arbitrary material particle b if, and only if, Equation (1d) is true.*

**Proof.**  Under the hypothesis $C_{i}=0$, Equation (31) reduces to

(32) $$\int_{b}\left(\dot{c}-H\right)db\;.$$

Due to the arbitrariness of the domain b and to the regularity of the integrand function, Equation (32) is equivalent to Equation (1d). □

According to second law of thermodynamics, the production of entropy along an arbitrary thermodynamic process, represented by a regular curve in the state space, cannot be negative. In the absence of external radiation (r=0), such a constraint can be expressed by the integral inequality

(33) $$\frac{d}{dt}\int_{b}\rho sdb+\int_{\partial b}J_{i}n_{i}d\sigma =\frac{d}{dt}\int_{b}\rho sdb+\int_{b}J_{i,i}db\geq 0\;,$$

where *s* is the internal entropy per unit of mass and $J_{i}$ is the local entropy flux through the boundary of b.

**Theorem** **5.** *The integral inequality (33) holds for an arbitrary material particle b if, and only if, the differential inequality (4) is true.*

**Proof.**  It is enough to observe that the integral inequality (33) can be reformulated as follows:

(34) $$\int_{b}\left(\rho \dot{s}+J_{i,i}\right)db\geq 0\;.$$

Due to the arbitrariness of the domain b and to the regularity of the integrand function, the global inequality (34) is equivalent to the local inequality (4). □

## 3. Thermodynamic Compatibility

In the present section, we explore the compatibility of Equation (1) with the second law of thermodynamics, namely with differential constraint (4). In order to better specify the theoretical framework of the present theory, we observe that the heat flux, whose evolution is ruled by Equation (1c), enters the state space. We observe also that the gradients of the independent thermodynamic variables belong to Σ. Such considerations allow to conclude that our model is within the frame of Extended Irreversible Thermodynamics (EIT), a weakly nonlocal thermodynamic theory in which the dissipative fluxes have the rank of independent thermodynamic variables [16,20,24,25,40]. According to the basic tenets of EIT, the gradients of the basic unknown fields can enter the state space.

When the dependency of *s* and $J_{i}$ on the state variables is made explicit, the inequality (4) reads

(35) $$\begin{array}{cc} \; & \varrho \left(\frac{\partial s}{\partial \varepsilon }\dot{\varepsilon }+\frac{\partial s}{\partial \varepsilon_{,i}}{\dot{\varepsilon }}_{,i}+\frac{\partial s}{\partial q_{i}}{\dot{q}}_{i}+\frac{\partial s}{\partial q_{j,i}}{\dot{q}}_{j,i}+\frac{\partial s}{\partial E_{ij}}{\dot{E}}_{ij}+\frac{\partial s}{\partial c}\dot{c}+\frac{\partial s}{\partial c_{,i}}{\dot{c}}_{,i}\right)+\\ \; & \frac{\partial J_{i}}{\partial \varepsilon }\varepsilon_{,i}+\frac{\partial J_{i}}{\partial \varepsilon_{,j}}\varepsilon_{,ji}+\frac{\partial J_{i}}{\partial q_{j}}q_{j,i}+\frac{\partial J_{i}}{\partial q_{j,k}}q_{j,ki}+\frac{\partial J_{i}}{\partial E_{hk}}E_{hk,i}+\frac{\partial J_{i}}{\partial c}c_{,i}+\frac{\partial J_{i}}{\partial c_{,k}}c_{,ki}\geq 0. \end{array}$$

Inequality (35) is exploited by a generalization of the classical Coleman–Noll procedure [17,18,19], which consists in substituting in (35) the evolution Equations (1b)–(1d) and their gradients up to the order of the gradients of ε, $q_{i}$ and *c* entering the state space (see also [41,42] for more details on the generalized exploitation procedures of the entropy inequality). Doing that, we consider of the first order of magnitude the gradients of the independent thermodynamic variables. Then, in a first-order approximation, we neglect the terms containing the products of such gradients as, for instance, the term $q_{h}E_{hk,i}q_{k,i}$. In this way, we obtain the following inequality:

(36) $$\begin{array}{cc} \; & \left(\frac{\partial s}{\partial q_{i}}\frac{\tau_{E}q_{j}}{\tau_{q}A_{q}}+\frac{\partial s}{\partial q_{i,k}}\tau_{E}q_{j,k}-\frac{\partial s}{\partial \varepsilon }T_{ij}+\varrho \frac{\partial s}{\partial E_{ij}}\right){\dot{E}}_{ij}+\\ \; & \left(\frac{\partial s}{\partial c_{,k}}\frac{\partial H}{\partial \varepsilon_{,i}}-\frac{\partial s}{\partial q_{i,k}}\frac{\kappa A_{K}}{\bar{c}\tau_{q}A_{q}}-\frac{\partial J_{i}}{\partial \varepsilon_{,k}}\right)\varepsilon_{,ki}+\\ \; & \left(\frac{\partial s}{\partial c_{,k}}\frac{\partial H}{\partial q_{j,i}}+\frac{\partial s}{\partial q_{i,k}}\frac{2\tau_{Q}q_{j}}{\tau_{q}\varrho \varepsilon A_{q}}-\frac{\partial s}{\partial \varepsilon_{,i}}\delta_{jk}+\frac{\partial J_{i}}{\partial q_{j,k}}\right)q_{j,ki}+\\ \; & \left(\frac{\partial J_{i}}{\partial E_{hk}}+\frac{\partial s}{\partial c_{,i}}\frac{\partial H}{\partial E_{hk}}\right)E_{hk,i}+\\ \; & \left(\frac{\partial s}{\partial q_{i,k}}\tau_{E}q_{j}-\frac{\partial s}{\partial \varepsilon_{,i}}T_{jk}\right){\dot{E}}_{ij,k}+\\ \; & \left(\frac{\partial s}{\partial c_{,k}}\frac{\partial H}{\partial c_{,i}}+\frac{\partial s}{\partial q_{i,k}}a_{c}+\frac{\partial J_{i}}{\partial c_{,k}}\right)c_{,ki}+f\left(\Sigma \right)\geq 0, \end{array}$$

wherein f(Σ) is a scalar-valued function defined on the state space whose expression is omitted for the sake of concision.

**Theorem** **6.** *Inequality (36) is satisfied whenever the thermodynamic process is if, and only if, the following thermodynamic restrictions hold:*

(37a) $$\begin{array}{cc} \; & \langle \frac{\partial s}{\partial q_{i}}\frac{\tau_{E}q_{j}}{\tau_{q}A_{q}}+\frac{\partial s}{\partial q_{i,k}}\tau_{E}q_{j,k}-\frac{\partial s}{\partial \varepsilon }T_{ij}+\varrho \frac{\partial s}{\partial E_{ij}}\rangle =0, \end{array}$$

(37b) $$\begin{array}{cc} \; & \langle \frac{\partial s}{\partial c_{,k}}\frac{\partial H}{\partial \varepsilon_{,i}}-\frac{\partial s}{\partial q_{i,k}}\frac{\kappa A_{K}}{\bar{c}\tau_{q}A_{q}}-\frac{\partial J_{i}}{\partial \varepsilon_{,k}}\rangle =0, \end{array}$$

(37c) $$\begin{array}{cc} \; & {\langle \frac{\partial s}{\partial c_{,k}}\frac{\partial H}{\partial q_{j,i}}+\frac{\partial s}{\partial q_{i,k}}\frac{2\tau_{Q}q_{j}}{\tau_{q}\varrho \varepsilon A_{q}}-\frac{\partial s}{\partial \varepsilon_{,i}}\delta_{jk}+\frac{\partial J_{i}}{\partial q_{j,k}}\rangle }_{\left(ki\right)}=0, \end{array}$$

(37d) $$\begin{array}{cc} \; & {\langle \frac{\partial J_{i}}{\partial E_{hk}}+\frac{\partial s}{\partial c_{,i}}\frac{\partial H}{\partial E_{hk}}\rangle }_{\left(hk\right)}=0, \end{array}$$

(37e) $$\begin{array}{cc} \; & {\langle \frac{\partial s}{\partial q_{i,k}}\tau_{E}q_{j}-\frac{\partial s}{\partial \varepsilon_{,i}}T_{jk}\rangle }_{\left(ij\right)}=0, \end{array}$$

(37f) $$\begin{array}{cc} \; & \langle \frac{\partial s}{\partial c_{,k}}\frac{\partial H}{\partial c_{,i}}+\frac{\partial s}{\partial q_{i,k}}a_{c}+\frac{\partial J_{i}}{\partial c_{,k}}\rangle =0, \end{array}$$

(37g) $$\begin{array}{cc} \; & f(\Sigma )\geq 0, \end{array}$$

*wherein symbol $\langle F\rangle$ denotes the symmetric part of the tensor function F, while symbol ${\langle F\rangle }_{{}_{\left(ab\ldots \right)}}$ denotes the symmetric part of F with respect to the indicated indices.*

**Proof.**  Preliminarily, let us evaluate inequality (36) in a fixed point $\bar{P}$ of the body at a fixed instant $\bar{t}$. Then, taking into account that, in a fixed point, the values of a function and those of its derivative are independent quantities, Equation (36) can be regarded as an algebraic inequality which is linear with respect to the elements of the set

(38) $$H\left(\bar{P},\bar{t}\right)\equiv \left\{\varepsilon_{,ki}\left(\bar{P},\bar{t}\right),c_{,ki}\left(\bar{P},\bar{t}\right),q_{j,ki}\left(\bar{P},\bar{t}\right),E_{hk,i}\left(\bar{P},\bar{t}\right),{\dot{E}}_{hk}\left(\bar{P},\bar{t}\right),{\dot{E}}_{hk,i}\left(\bar{P},\bar{t}\right)\right\}$$

called the set of the higher derivatives in $\left(\bar{P},\bar{t}\right)$. Such derivatives are independent of the quantities in the brackets, which, instead, depend on the elements of

$$\Sigma \left(\bar{P},\bar{t}\right)\equiv \left\{\varepsilon \left(\bar{P},\bar{t}\right),\varepsilon_{,k}\left(\bar{P},\bar{t}\right),c\left(\bar{P},\bar{t}\right),c_{,k}\left(\bar{P},\bar{t}\right),q_{h}\left(\bar{P},\bar{t}\right),q_{h,k}\left(\bar{P},\bar{t}\right),E_{hk}\left(\bar{P},\bar{t}\right)\right\}.$$

Moreover, since inequality (36) must hold for arbitrary thermodynamic processes, the elements of $H(\bar{P},\bar{t})$ can have arbitrary sign and, consequently, the same is true for each of the linear terms entering such inequality. This implies that the inequality can be violated for some values of the elements of $H(\bar{P},\bar{t})$, unless all the linear terms in it vanish. This happens if, and only if, the thermodynamic restrictions (37) hold in $\left(\bar{P},\bar{t}\right)$, once the symmetry of the elements of $H(\bar{P},\bar{t})$ is taken into account. On the other hand, since the point $\left(\bar{P},\bar{t}\right)$ is arbitrary, thermodynamic restrictions (37) ensue. □

**Remark** **2.** *Inequality (37g) is also called reduced-entropy inequality since it represents the effective local rate of entropy production once restrictions (37a)–(37f) have been satisfied.*

**Remark** **3.** *The classical Coleman–Noll procedure [17] consists in substituting in Equation (35) the sole evolution Equations (1b)–(1d). Thus, the set of the higher derivatives becomes*

$$H\left(\bar{P},\bar{t}\right)\equiv \left\{\varepsilon_{,ki}\left(\bar{P},\bar{t}\right),c_{,ki}\left(\bar{P},\bar{t}\right),q_{j,ki}\left(\bar{P},\bar{t}\right),E_{hk,i}\left(\bar{P},\bar{t}\right),{\dot{E}}_{hk}\left(\bar{P},\bar{t}\right),{\dot{E}}_{hk,i}\left(\bar{P},\bar{t}\right),{\dot{\varepsilon }}_{,i}\left(\bar{P},\bar{t}\right),{\dot{c}}_{,k}\left(\bar{P},\bar{t}\right),{\dot{q}}_{i,k}\left(\bar{P},\bar{t}\right)\right\},$$

*and the set of coefficients which must vanish is different with respect to the previous one. In particular, according to such a procedure, the coefficients of the quantities ${\dot{\varepsilon }}_{,i}$, ${\dot{c}}_{,k}$, and ${\dot{q}}_{i,k}$ must be zero, preventing so the dependency of s on $\varepsilon_{,i}$, $c_{,k}$, and $q_{i,k}$.*

*It is expected that such a methodology implies more severe restrictions on the constitutive quantities because it requires that the entropy inequality is satisfied under constraints (1b)–(1d), independently of the form of the evolution equations of the quantities ${\dot{\varepsilon }}_{,i}$, ${\dot{c}}_{,k}$, and ${\dot{q}}_{i,k}$. It is very efficient in those physical situations in which it is expected that the partial derivatives of s with respect to the gradients of the unknown fields vanish. However, for some systems as, for instance, Korteweg fluids [18,19], such derivatives should not vanish, so that the generalized method seems to be more suited for the exploitation of the entropy inequality. Below, we analyze some cases for the system at hand in which s should depend on the gradients. The generalized Coleman–Noll method reduces to the classical one if the form of the gradients of the unknown fields is not taken into account.*

**Corollary** **1.** *If inequality (35) is restricted by sole constraints (1b)–(1d), the following thermodynamic restrictions hold:*

(39a) $$\begin{array}{cc} \; & s=s(\varepsilon ,c,q_{i},E_{ij}), \end{array}$$

(39b) $$\begin{array}{cc} \; & {\langle \frac{\partial s}{\partial q_{i}}\frac{\tau_{E}q_{j}}{\tau_{q}A_{q}}-\frac{\partial s}{\partial \varepsilon }T_{ij}+\varrho \frac{\partial s}{\partial E_{ij}}\rangle }_{\left(ij\right)}=0, \end{array}$$

(39c) $$\begin{array}{cc} \; & \langle \frac{\partial J_{i}}{\partial \varepsilon_{,k}}\rangle =0, \end{array}$$

(39d) $$\begin{array}{cc} \; & {\langle \frac{\partial J_{i}}{\partial q_{j,k}}\rangle }_{\left(ki\right)}=0, \end{array}$$

(39e) $$\begin{array}{cc} \; & {\langle \frac{\partial J_{i}}{\partial E_{hk}}\rangle }_{\left(hk\right)}=0, \end{array}$$

(39f) $$\begin{array}{cc} \; & \langle \frac{\partial J_{i}}{\partial c_{,k}}\rangle =0, \end{array}$$

(39g) $$\begin{array}{cc} \; & g(\Sigma )\geq 0, \end{array}$$

*wherein g is a new scalar-valued function which can be obtained by f once quantities $\frac{\partial s}{\partial \varepsilon_{,i}}$, $\frac{\partial s}{\partial q_{i,k}}$, and $\frac{\partial s}{\partial c_{,k}}$ are put equal to zero.*

**Proof.**  To prove the Corollary, it is enough to observe that, without the substitution of the gradient extensions of Equations (1b)–(1d) into entropy inequality (35), the terms $\frac{\partial s}{\partial \varepsilon_{,i}}{\dot{\varepsilon }}_{,i}$, $\frac{\partial s}{\partial q_{i,k}}{\dot{q}}_{i,k}$, and $\frac{\partial s}{\partial c_{,k}}{\dot{c}}_{,k}$ would not couple with any other term. This would imply the following thermodynamic restrictions:

(40a) $$\begin{array}{cc} \; & \frac{\partial s}{\partial \varepsilon_{,i}}=0, \end{array}$$

(40b) $$\begin{array}{cc} \; & \frac{\partial s}{\partial q_{i,k}}=0, \end{array}$$

(40c) $$\begin{array}{cc} \; & \frac{\partial s}{\partial c_{,k}}c_{,k}=0, \end{array}$$

i.e, $s=s(\varepsilon ,c,q_{i},E_{ij})$. The coupling of Equations (37) and (40) yields Equations (39b)–(39g). □

**Corollary** **2.** *In order to satisfy system of restrictions (39), it is sufficient that the following set of relations is true:*

(41a) $$\begin{array}{cc} \; & s=s(\varepsilon ,c,q_{i},E_{ij}), \end{array}$$

(41b) $$\begin{array}{cc} \; & \frac{\partial s}{\partial q_{i}}\frac{\tau_{E}q_{j}}{\tau_{q}A_{q}}-\frac{\partial s}{\partial \varepsilon }T_{ij}+\varrho \frac{\partial s}{\partial E_{ij}}=0, \end{array}$$

(41c) $$\begin{array}{cc} \; & J_{i}=J_{i}\left(\varepsilon ,c,q_{i}\right), \end{array}$$

(41d) $$\begin{array}{cc} \; & h(\Sigma )\geq 0, \end{array}$$

*where h is a new function which can be obtained by g once quantities $\frac{\partial J_{i}}{\partial \varepsilon_{,k}}$, $\frac{\partial J_{i}}{\partial q_{j,k}}$, $\frac{\partial J_{i}}{\partial E_{hk}}$, and $\frac{\partial J_{i}}{\partial c_{,k}}$ are put equal to zero.*

**Proof.**  It is evident that if Equation (41b) is true, then restriction (39b) is satisfied. Moreover, if $\frac{\partial J_{i}}{\partial \varepsilon_{,k}}=0$, $\frac{\partial J_{i}}{\partial q_{j,k}}=0$, $\frac{\partial J_{i}}{\partial E_{hk}}=0$, and $\frac{\partial J_{i}}{\partial c_{,k}}=0$, i.e., if Equation (41c) is true, then restrictions (39c)–(39f) hold, while function *g* in Equation (39g) reduces to function *h* in Equation (41d). □

**Corollary** **3.** *Thermodynamic restrictions (41a)–(41c) are satisfied by the following constitutive equations for the specific entropy, the Cauchy stress and the entropy flux:*

(42a) $$\begin{array}{cc} \; & s=s_{0}\left(\varepsilon ,\bar{c}\right)+\frac{s_{1}\bar{c}}{2\varepsilon }q_{i}^{2}+\frac{\bar{c}}{\rho \varepsilon }\left[\frac{\lambda }{2}E_{ii}^{2}+\mu E_{ij}^{2}-\left(3\lambda +2\mu \right)\frac{\alpha }{\bar{c}}\varepsilon E_{ij}\delta_{ij}\right], \end{array}$$

(42b) $$\begin{array}{cc} \; & T_{ij}=\frac{s_{1}\tau_{E}q_{i}q_{j}}{\tau_{q}A_{q}}+\lambda E_{hh}\delta_{ij}+2\mu E_{ij}-\left(3\lambda +2\mu \right)\frac{\alpha }{\bar{c}}\varepsilon \delta_{ij}, \end{array}$$

(42c) $$\begin{array}{cc} \; & J_{i}=\frac{\bar{c}}{\varepsilon }q_{i}, \end{array}$$

*wherein $s_{0}$ and $s_{1}$ are material functions depending on ε and c, λ and μ are the Lam$\overset{´}{e}$ coefficients, and α is the coefficient of thermal expansion.*

**Proof.**  It is evident that constitutive Equation (42a) satisfies restriction (41a), and constitutive Equation (42c) satisfies restriction (41c). Moreover, by Equation (42a), we obtain

(43a) $$\begin{array}{cc} \; & \frac{\partial s}{\partial q_{i}}=\frac{s_{1}\bar{c}}{\varepsilon }q_{i}, \end{array}$$

(43b) $$\begin{array}{cc} \; & \frac{\partial s}{\partial E_{ij}}=\frac{\bar{c}}{\rho \varepsilon }\left[\lambda E_{hh}\delta_{ij}+2\mu E_{ij}-\left(3\lambda +2\mu \right)\frac{\alpha }{\bar{c}}\varepsilon \delta_{ij}\right]. \end{array}$$

By Equations (43a) and (43b), it follows immediately that restriction (41b) is satisfied once the thermodynamic relation $\frac{\partial s}{\partial \varepsilon }=\frac{1}{\vartheta }=\frac{\bar{c}}{\varepsilon }$ is taken into account. □

**Remark** **4.** *Equation (42) yields the classical expression of the entropy flux postulated by Coleman and Noll in their celebrated paper on the thermodynamics of viscoelastic heat conducting solids [17], while Equations (42a) and (42b) generalize the constitutive equations for s and $T_{ij}$ obtained in classical linear thermoelasticity [43]. Both constitutive Equations (42a) and (42b) are local, as expected, and represent a very particular case of the more general constitutive equations compatible with the set of thermodynamic restrictions in Equation (37).*

**Remark** **5.** *It is important to observe that the material coefficients λ, μ, and α, entering Equations (42a) and (42b), depend, in general, on ε and c.*

**Remark** **6.** *Due to the presence in s of the term $\frac{s_{1}\bar{c}}{2\varepsilon }q_{i}^{2}$, in nonequilibrium situations the temperature $\vartheta ={\left(\frac{\partial s}{\partial \varepsilon }\right)}^{-1}$ depends on the heat flux, too.*

One could wonder if, for the system at hand, the application of the generalized exploitation procedure is necessary or not. To answer that question, we observe preliminarily that, since function *H* depends on $E_{ij}$, the gradient of *c* is not constant in time, as in the case of rigid heat conductors, but can change as a consequence of the deformation, as it can be easily seen by taking the gradient of Equation (1d). This variable gradient contributes to the variation of the heat flux, too, according to Equation (1c), and this produces further thermal dissipation. Thus, both *s* and $J_{i}$ are expected to contain terms which depend on $E_{ij}$ and $c_{,i}$. Moreover, by Equation (1c), it follows that the temperature varies along the medium even in the absence of the heat flux, so that a dependency of *s* and $J_{i}$ on $\varepsilon_{,i}$ is expected as well. The propositions below clarify the conditions under which such dependencies are possible. To this end, we generalize Equation (42c) as follows:

(44) $$J_{i}=\frac{\bar{c}}{\varepsilon }q_{i}+J_{0}\left(\varepsilon ,c,E_{ij}\right)\varepsilon_{,i}+J_{1}\left(\varepsilon ,c,E_{ij}\right)c_{,i},$$

and investigate its compatibility with the second law of thermodynamics.

**Corollary** **4.** *The constitutive equation of $J_{i}$ may depend on $E_{ij}$ if, and only if, s depends on $c_{,i}$ and H depends on $E_{ij}$.*

**Proof.**  To prove this proposition, we observe that if either *s* is independent of $c_{,i}$ or *H* is independent of $E_{ij}$, thermodynamic restriction (37d) implies

${\langle \frac{\partial J_{i}}{\partial E_{hk}}\rangle }_{\left(hk\right)}=0$. On the other hand, since strain tensor $E_{hk}$ is symmetric, this relation is equivalent to $\frac{\partial J_{i}}{\partial E_{hk}}=0$, i.e., $J_{i}$ is independent of $E_{hk}$. □

**Corollary** **5.** *The constitutive equation of $J_{i}$ may be linear in $\varepsilon_{,k}$ (as in Equation (44)) if, and only if, either s depends on $c_{,i}$ and H depends on $\varepsilon_{,i}$ or s depends on $q_{i,k}$.*

**Proof.**  To prove this proposition, it is enough to observe that if *s* is independent of $c_{,i}$, *H* is independent of $\varepsilon_{,i}$, and *s* in independent of $q_{i,k}$, then thermodynamic restriction (37b) implies $\langle \frac{\partial J_{i}}{\partial \varepsilon_{,k}}\rangle =0$ or, equivalently, $\frac{1}{2}\left(\frac{\partial J_{i}}{\partial \varepsilon_{,k}}+\frac{\partial J_{k}}{\partial \varepsilon_{,i}}\right)=0$. It yields $J_{0}\delta_{ik}=0$ once Equation (44) is taken into account. □

**Corollary** **6.** *The constitutive equation of $J_{i}$ may be linear in $c_{,k}$ (as in Equation (44)) if, and only if, either s depends on $c_{,k}$ and H depends on $c_{,i}$ or s depends on $q_{i,k}$.*

**Proof.**  To prove this proposition, it is enough to observe that if *s* is independent of $c_{,k}$ or *H* is independent of $\varepsilon_{,i}$, and *s* in independent of $q_{i,k}$, then thermodynamic restriction (37f) implies $\langle \frac{\partial J_{i}}{\partial c_{,k}}\rangle =0$ or, equivalently, $\frac{1}{2}\left(\frac{\partial J_{i}}{\partial c_{,k}}+\frac{\partial J_{k}}{\partial c_{,i}}\right)=0$. It yields $J_{1}\delta_{ik}=0$ once Equation (44) is taken into account. □

The nonlocal terms entering Equation (44) may be important at a nanometric scale, where nonlocal effects are evident. One should note that the additional terms $J_{0}\left(\vartheta ,c,E_{ij}\right)\varepsilon_{,i}+J_{1}\left(\vartheta ,c,E_{ij}\right)c_{,i}$ in Equation (44) may be regarded as the entropy extraflux proposed in [44].

## 4. Thermoelastic-Wave Propagation

In this section, we study the propagation of thermoelastic waves along a graded material. Our analysis is pursued under the following hypotheses:

- constitutive Equation (42b) for the Cauchy stress holds;
- constitutive Equation (30) for the flux of heat flux, leading to the balance Equation (1c) for the heat flux, holds;
- all the material coefficients are constant;
- function *H* is linear in the gradients, i.e., the constitutive equation for *H* is
  (45) $$H=H_{0}\left(\varepsilon ,E_{hk},c\right)+h_{k}\varepsilon_{,k}+m_{k}c_{,k}+N_{kj}q_{k,j},$$
  wherein $h_{k}$, $m_{k}$ and $N_{kj}$ depend on set $\left(\varepsilon ,E_{hk},c\right)$;
- $\frac{\partial \left(s_{1}\tau_{E}/\tau_{q}A_{q}\right)}{\partial x_{j}}$ is negligible.

Under the hypotheses above, the system of Equation (1) writes as
  (46a) $$\begin{array}{cc} \; & \varrho {\ddot{u}}_{i}-\frac{s_{1}\tau_{E}q_{i,j}q_{j}}{\tau_{q}A_{q}}-\frac{s_{1}\tau_{E}q_{i}q_{j,j}}{\tau_{q}A_{q}}-\lambda E_{hh,i}-2\mu E_{ij,j}+\bar{b}\varepsilon_{,i}=0, \end{array}$$
  (46b) $$\begin{array}{cc} \; & \varrho \dot{\varepsilon }-\frac{s_{1}\tau_{E}q_{i}q_{j}}{\tau_{q}A_{q}}{\dot{E}}_{ij}-\lambda E_{hh}{\dot{E}}_{ii}-2\mu E_{ij}{\dot{E}}_{ij}+\bar{b}\varepsilon {\dot{E}}_{ii}+q_{i,i}=0, \end{array}$$
  (46c) $$\begin{array}{cc} \; & \tau_{q}A_{q}{\dot{q}}_{i}+q_{i}+\bar{\kappa }A_{K}\varepsilon_{,i}-\frac{2\tau_{Q}}{\varrho \varepsilon }q_{j,i}q_{j}-\tau_{E}{\dot{E}}_{ij}q_{j}-a_{c}c_{,i}=0, \end{array}$$
  (46d) $$\begin{array}{cc} \; & \dot{c}=H_{0}\left(\varepsilon ,E_{hk},c\right)+h_{k}\varepsilon_{,k}+m_{k}c_{,k}+N_{kj}q_{k,j}, \end{array}$$
  with $\bar{b}=\left(3\lambda +2\mu \right)\alpha /\bar{c}$, and $\bar{\kappa }=\kappa /\bar{c}$.

**Remark** **7.** *It is evident that by the results in Section 3, it follows that constitutive Equations (42b), (30) and (45) are compatible with the second law of thermodynamics.*

The nonlinear system of equations above allows the existence of nonregular solutions.

**Definition** **1.** *An acceleration wave is a traveling surface S across which solution $\left\{u_{i},\varepsilon ,q_{i},c\right\}$ of Equations (46) is continuous, but its first- and higher-order derivatives suffer jump discontinuities [26,45].*

**Remark** **8.** *It is worth observing that Equations (46) hold in the points of the two regions behind and ahead S, while on S those equations must be written in terms of the jumps of the discontinuous fields across the wavefront. Such jumps are given by differences*

(47) $$\begin{array}{cccc} \Delta {\ddot{u}}_{i}={{\ddot{u}}_{i}}^{-}-{{\ddot{u}}_{i}}^{+}, & \Delta u_{i,j}=u_{i,j}^{-}-u_{i,j}^{+}, & \Delta u_{i,jk}=u_{i,jk}^{-}-u_{i,jk}^{+}, & \\ \Delta \varepsilon_{,j}=\varepsilon_{,j}^{-}-\varepsilon_{,j}^{+}, & \Delta q_{i,j}=q_{i,j}^{-}-q_{i,j}^{+}, & \Delta c_{,j}=c_{,j}^{-}-c_{,j}^{+}, \end{array}$$

*where upscript + denotes the value of the corresponding fields in the region which S is about to enter, and upscript − denotes the same value in the region which S is about to leave.*

For the sake of simplicity, herein we consider a one-dimensional system. Then, Equations (46) write as

(48a) $$\begin{array}{cc} \; & \varrho \ddot{u}-\left(\lambda +2\mu \right)u_{,xx}+\bar{b}\varepsilon_{,x}-2\frac{s_{1}\tau_{E}}{\tau_{q}A_{q}}qq_{,x}=0, \end{array}$$

(48b) $$\begin{array}{cc} \; & -\frac{s_{1}\tau_{E}q^{2}}{\tau_{q}A_{q}}{\dot{u}}_{,x}-\left(\lambda +2\mu \right)u_{,x}{\dot{u}}_{,x}+\bar{b}\varepsilon {\dot{u}}_{,x}+\varrho \dot{\varepsilon }+q_{,x}=0, \end{array}$$

(48c) $$\begin{array}{cc} \; & -\tau_{E}q{\dot{u}}_{,x}+\bar{\kappa }A_{K}\varepsilon_{,x}+\tau_{q}A_{q}\dot{q}+q-\frac{2\tau_{Q}}{\varrho \varepsilon }qq_{,x}-a_{c}c_{,x}=0, \end{array}$$

(48d) $$\begin{array}{cc} \; & H_{0}+h\varepsilon_{,x}+Nq_{,x}-\dot{c}+mc_{,x}=0. \end{array}$$

In order to determine the jumps of the discontinuous fields across S, we first observe that in the one-dimensional case, the sole component of the strain tensor is $E=u_{,x}$. Here and in the following, we suppose that fields $u_{,x},\;\varepsilon ,\;q,\;c$ are continuous across S but their space and time derivatives suffer jump discontinuities. In the meantime, we suppose that across S, the time derivatives of displacement *u* are discontinuous, too. Hence, we introduce the following notation:

(49) $$\delta \frac{\partial^{2}u}{\partial x^{2}}=\delta \frac{\partial (\partial u/\partial x)}{\partial x}=\delta \frac{\partial E}{\partial x}\equiv \delta E,\;\;\;\;\;\;\delta \frac{\partial \varepsilon }{\partial x}\equiv \delta \varepsilon ,\;\;\;\;\;\;\delta \frac{\partial q}{\partial x}\equiv \delta q,\;\;\;\;\;\;\delta \frac{\partial c}{\partial x}\equiv \delta c,$$

with δ as the jump of the indicated quantities in the onedimensional case. Moreover, $u_{,x}$ being continuous across S, by the classical Hadamard identities (see Ref. [45] and Equations (54) and (55) therein), we can write

(50) $$\delta \frac{\partial^{2}u}{\partial t^{2}}=U^{2}\delta \frac{\partial^{2}u}{\partial x^{2}}=U^{2}\delta E,$$

wherein *U* is the speed of propagation of thermomechanical disturbances. Then, system (48) yields

(51a) $$\begin{array}{cc} \; & \left[\varrho U^{2}-\left(\lambda +2\mu \right)\right]\;\delta E+\bar{b}\;\delta \varepsilon -2\frac{s_{1}\tau_{E}}{\tau_{q}A_{q}}q\;\delta q=0, \end{array}$$

(51b) $$\begin{array}{cc} \; & U[\frac{s_{1}\tau_{E}q^{2}}{\tau_{q}A_{q}}+\left(\lambda +2\mu \right)E-\bar{b}\varepsilon ]\;\delta E-\varrho U\;\delta \varepsilon +\delta q=0, \end{array}$$

(51c) $$\begin{array}{cc} \; & U\tau_{E}q\;\delta E+\bar{\kappa }A_{K}\;\delta \varepsilon -\left[U\tau_{q}A_{q}+\frac{2\tau_{Q}}{\varrho \varepsilon }q\right]\;\delta q-a_{c}\;\delta c=0, \end{array}$$

(51d) $$\begin{array}{cc} \; & h\;\delta \varepsilon +N\;\delta q+(U+m)\delta c=0, \end{array}$$

wherein it must be understood that the coefficients of the unknown jumps are evaluated in the region which S is about to enter. Equations (50) represent a linear and homogeneous algebraic system in the unknown quantities δE, δε, δq and δc, which provides, in principle, the values of the jumps we are looking for. The following statement is straightforward.

**Theorem** **7.** *System (51) admits nontrivial solution if, and only if, the following condition is fulfilled:*

(52) $$det\left[\begin{array}{cccc} \left[\varrho U^{2}-\left(\lambda +2\mu \right)\right] & \bar{b} & -2\frac{s_{1}\tau_{E}}{\tau_{q}A_{q}}q & 0\\ U[\frac{s_{1}\tau_{E}q^{2}}{\tau_{q}A_{q}}+\left(\lambda +2\mu \right)E-\bar{b}\varepsilon ] & -\varrho U & 1 & 0\\ U\tau_{E}q & \bar{\kappa }A_{K}\;\;\;\; & -(U\tau_{q}A_{q}+\frac{2\tau_{Q}}{\varrho \varepsilon }q)\;\;\;\; & -a_{c}\\ 0 & h & N & \left(U+m\right) \end{array}\right]=0.$$

**Proof.**  The proof immediately follows by the observation that system (51) admits non-trivial solutions if, and only if, the matrix of the coefficients is singular, i.e., if, and only if, Equation (52) holds. □

We suppose now that the following additional conditions hold:

(53) $$\tau_{E}=\tau_{Q}=N=\bar{b}=0.$$

**Remark** **9.** *Conditions (53) mean that the Cauchy stress coincides with that of classical linear elasticity in the absence of thermal effects. Moreover, nonlocal effects for the heat flux do not influence the evolution equations for the heat flux (Equation (1c)) and for c (Equation (1d)).*

Under hypotheses (53), system (51) becomes

(54a) $$\begin{array}{cc} \; & [\varrho U^{2}-\left(\lambda +2\mu \right)]\;\delta E=0, \end{array}$$

(54b) $$\begin{array}{cc} \; & U(\lambda +2\mu )E\;\delta E-\varrho U\;\delta \varepsilon +\delta q=0, \end{array}$$

(54c) $$\begin{array}{cc} \; & \bar{\kappa }A_{K}\;\delta \varepsilon -U\tau_{q}A_{q}\;\delta q-a_{c}\;\delta c=0, \end{array}$$

(54d) $$\begin{array}{cc} \; & h\;\delta \varepsilon +(U+m)\delta c=0. \end{array}$$

**Theorem** **8.** *System (54) admits nontrivial solution if, and only if, the following equation is fulfilled:*

(55) $$\left[\varrho U^{2}-\left(\lambda +2\mu \right)\right]\left[\varrho U^{2}\left(U\tau_{q}A_{q}+m\tau_{q}A_{q}\right)-\left(\left(U+m\right)\bar{\kappa }A_{K}+ha_{c}\right)\right]=0.$$

**Proof.**  To prove the theorem, we observe that system (54) admits non-trivial solutions if, and only if,

(56) $$det\left[\begin{array}{cccc} \left[\varrho U^{2}-\left(\lambda +2\mu \right)\right] & 0 & 0 & 0\\ U(\lambda +2\mu )E & -\varrho U & 1 & 0\\ 0 & \bar{\kappa }A_{K}\;\;\;\; & -U\tau_{q}A_{q}\;\;\;\; & -a_{c}\\ 0 & h & 0 & \left(U+m\right) \end{array}\right]=0,$$

i.e, if, and only if, Equation (55) holds. □

**Remark** **10.** *Compatibility condition (55) is satisfied if either two elastic waves propagate with speeds*

(57) $$U=\pm \sqrt{\frac{\lambda +2\mu }{\varrho }}\;\;\;\;\;\;\;\;$$

*or two thermoelastic waves propagate with speeds*

(58) $$U=-m,\;\;\;\;\;\;U=-m-\frac{ha_{c}}{\bar{\kappa }A_{K}}.$$

**Remark** **11.** *If all the relaxation times in Equation (1c) vanish, then the generalized Fourier law*

(59) $$q_{i}=-\bar{\kappa }A_{K}\varepsilon_{,i}-a_{c}c_{,i}$$

*holds. Thus, as $q_{i}=q_{i}\left(\varepsilon_{,i},\;c_{,i},\;E_{hk}\right)$, we are in the realm of Rational Thermodynamics, i.e., the thermodynamic theory in which heat flux and stress tensor are assigned through suitable constitutive Equations [16,31].*

**Corollary** **7.** *Under the validity of the generalized Fourier law (59), if*

(60) $$N=\bar{b}=0,$$

*then the following speeds of propagation are possible:*

(61) $$U=\pm \sqrt{\frac{\lambda +2\mu }{\varrho }},\;\;\;\;\;U=-m-\frac{ha_{c}}{\bar{\kappa }A_{K}}.$$

**Proof.**  The proof follows by the observation that, if $\tau_{q}=0$, Equation (55) reduces to

(62) $$\left[\varrho U^{2}-\left(\lambda +2\mu \right)\right]\left[\left(U+m\right)\bar{\kappa }A_{K}+ha_{c}\right]=0.$$

□

**Remark** **12.** *The first speed in Equation (61) is of elastic type, while the second one, due to the combined presence of $\bar{\kappa }$ and $A_{K}$, corresponds to a thermo-mechanical wave. If $a_{c}=0$, then the evolution equation of the heat flux does not appear in the wave speeds, and the constant speed U=−m corresponds to a stoichiometric wave, i.e., to a transport of energy due to the variable composition.*

**Corollary** **8.** *Under hypotheses (53), if*

(63) $$a_{c}=m=0,\;\;\;A_{q}≃1,\;\;\;A_{K}≃1,$$

*the propagation of elastic or thermal perturbations with speeds*

(64) $$U=\pm \sqrt{\frac{\lambda +2\mu }{\varrho }},\;\;\;\;\;U=\pm \sqrt{\frac{\bar{\kappa }}{\varrho \tau_{q}}}$$

*is possible*.

**Proof.**  We first observe that hypotheses (53) imply Equation (55). On the other hand, if conditions (63) hold, then Equation (55) reduces to

(65) $$\left[\varrho U^{2}-\left(\lambda +2\mu \right)\right]\left[U^{2}\varrho \tau_{q}-\bar{\kappa }\right]=0,$$

which proves the assertion. □

**Remark** **13.** *Under the hypotheses of Corollary 8, the system behaves as a purely elastic body (no thermoelastic coupling), with a Cattaneo-type evolution equation for the heat flux. Thus, the presence of purely elastic and purely thermal waves (second sound) is expected.*

**Definition** **2.** *An acceleration wave is said to propagate in a state of thermal equilibrium if in the region in which the wave is about to enter*

(66) $$\dot{\varepsilon }=\varepsilon_{,x}=q=0.$$

In such a case, system (51) becomes

(67a) $$\begin{array}{cc} \; & \left[\varrho U^{2}-\left(\lambda +2\mu \right)\right]\;\delta E+\bar{b}\;\delta \varepsilon =0, \end{array}$$

(67b) $$\begin{array}{cc} \; & U[\left(\lambda +2\mu \right)E-\bar{b}\varepsilon ]\;\delta E-\varrho U\;\delta \varepsilon +\delta q=0, \end{array}$$

(67c) $$\begin{array}{cc} \; & \bar{\kappa }A_{K}\;\delta \varepsilon -U\tau_{q}A_{q}\;\delta q-a_{c}\;\delta c=0, \end{array}$$

(67d) $$\begin{array}{cc} \; & h\;\delta \varepsilon +N\;\delta q+(U+m)\delta c=0. \end{array}$$

**Theorem** **9.** *System (67) admits nontrivial solution if, and only if, the following condition is fulfilled:*

(68) $$det\left[\begin{array}{cccc} \left[\varrho U^{2}-\left(\lambda +2\mu \right)\right] & \bar{b} & 0 & 0\\ U[\left(\lambda +2\mu \right)E-\bar{b}\varepsilon ] & -\varrho U & 1 & 0\\ 0 & \bar{\kappa }A_{K}\;\;\;\; & -U\tau_{q}A_{q},\;\;\; & -a_{c}\\ 0 & h & N & \left(U+m\right) \end{array}\right]=0.$$

**Proof.**  The proof immediately follows by the observation that system (67) admits non-trivial solutions if, and only if, the matrix of the coefficients is singular, i.e., if, and only if, Equation (68) holds. □

**Corollary** **9.** *If m=0, system (65) admits a non-trivial solution if, and only if, the following condition is fulfilled:*

(69) $$\left[\varrho U^{2}-\left(\lambda +2\mu \right)\right]\left[\varrho U^{3}\tau_{q}A_{q}-U\left(\varrho Na_{c}+\bar{\kappa }A_{K}\right)+ha_{c}\right]-\bar{b}U\left[\left(\lambda +2\mu \right)E-\bar{b}\varepsilon \right]\left[-U^{2}\tau_{q}A_{q}+Na_{c}\right]=0.$$

**Proof.**  To prove this statement, we observe that Equation (69) follows by Equation (68) once condition m=0 is taken into account. □

**Remark** **14.** *Equation (69) is satisfied if two elastics of speed*

(70) $$U=\pm \sqrt{\frac{\lambda +2\mu }{\varrho }}$$

*propagate together with two thermoelastic waves with speed*

(71) $$U=\pm \sqrt{\frac{Na_{c}}{\tau_{q}A_{q}}}.$$

*Moreover, if the third-grade equation*

(72) $$\varrho U^{3}\tau_{q}A_{q}-U\left(\varrho Na_{c}+\bar{\kappa }A_{K}\right)+ha_{c}=0$$

*admits real solutions, then Equation (69) is also satisfied if thermoelastic waves with speed given by such solutions propagate together with two thermoelastic waves with speed given by Equation (71).*

## 5. Conclusions

In the last decades, substantial effort was dedicated to fabricating functionally graded materials, with the scope of controlling their physical properties such as heat conduction, electric conductivity and resistance to enhanced stresses. Such investigations ranged over a characteristic length scale from nanometers to millimeters [13]. A functionally graded material is a material with graded functions inside them. In fact, an ordinary composite material contains a sudden change in properties at some interfaces, while a functionally graded material presents a gradual change inside it. This leads to graded patterns of material composition and/or microstructures. Modeling graded patterns are complex and need incorporating the material property gradient at a very small scale, thus reducing the discontinuity of the material distribution. Such systems exhibit many advantages compared to conventional alloys and composite materials since they provide means for controlling material response to deformation and dynamic loading. In addition, biocompatibility of many of them increases their suitability for medical applications.

Another important aspect regards the fabrication of ultra-high-temperature resistant materials for aircrafts, space vehicles and other engineering applications. Such heat-resistant materials contain reinforcing particles (ceramics) in a metallic matrix, with the metallic matrix subjected to plastic deformation and generation of cracks. In such a case, an accurate analysis of the evolution of thermal stresses is important. However, this goal cannot be achieved without considering the law of variation of the internal microstructure, too.

Thus, the study of elastic and thermal properties of composition graded systems is a promising field of research, since applied mechanical and thermal stresses may influence their thermal conductivity, thus providing a wider degree of control of their behavior [8,14,15].

In Equation (1), we proposed a mathematical model describing the nonlinear thermoelastic behavior of composition graded materials. The compatibility of the aforementioned model with the second law of thermodynamics was investigated by applying a generalized Coleman–Noll procedure.

We showed that for the material at hand, the specific entropy and the stress tensor may depend on the gradient of the unknown fields, resulting in a very general theory. The speeds of propagation of coupled first- and second-sound pulses, propagating either in non-equilibrium or in equilibrium states, were calculated under different physical conditions. We found that several different types of thermomechanical perturbations may propagate, depending on the value of the material coefficients characterizing the system.

The basic assumption underlying the present work is that the deformation of the body can produce changes in the stoichiometry, altering locally the material composition. Although such assumption is only based on theoretical considerations (see Section 1), in future research, it can be tested by comparing the results on the speeds of thermomechanical disturbances shown in Section 4 with numerical simulation of wave propagation (see, for instance, Ref. [30]). The usefulness of the present analysis becomes more evident if one considers that the variation with respect to time of c can be established in the phase of fabrication of the graded material in order to optimize its performance. The novelties of the present research are represented by the fully nonlocal constitutive equations, by the generalized exploitation procedure of the entropy inequality, which allows the specific entropy and the stress tensor to depend on the gradient of the unknown fields, and by the propagation of several different types of coupled first- and second-sound pulses, depending on the value of the material coefficients. An interesting propagation is that of pure stoichiometric waves, with speed U=−m where *m* measures the nonlocality of $\dot{c}$ with respect to *c* itself (see Equation (46d)). To the best of our knowledge, this is the first time that such a type of propagation has been obtained. The coupling of thermal and stoichiometric effects was taken into account through the term $-a_{c}c_{,i}$ in the evolution equation of the heat flux (see Equation (1d)). Such a coupling produces thermoelastic waves with speed

(73) $$U=-m-\frac{ha_{c}}{\bar{\kappa }A_{K}},$$

for propagation in nonequilibrium states and

(74) $$U=\pm \sqrt{\frac{Na_{c}}{\tau_{q}A_{q}}},$$

for propagation in equilibrium states. Furthermore, it is capable to produce additional thermoelastic waves with speed given by the real solutions of Equation (72). It should be noted that if $a_{c}=0$, besides solution U=0, Equation (72) yields

(75) $$U=\pm \sqrt{\frac{\bar{\kappa }A_{K}}{\varrho \tau_{q}A_{q}}},$$

which corresponds to heat waves with strain-dependent thermal conductivity. Finally, the model is capable to reproduce the propagation of pure elastic waves and pure thermal waves in the absence of coupling (see Equation (64)). Such a rich behavior was obtained under hypothesis $\tau_{Q}=\tau_{E}=0$.

As a concluding remark, let us compare the results obtained in the present paper with those obtained in [4,5], wherein a constitutive theory of composition graded materials has been developed as well. In [4], stoichiometric variable *c* enters the state space, but not its gradient. An evolution equation of type Equation (1c) for the heat flux is postulated. However, no governing equation for the evolution of *c* has been postulated. Hence, stoichiometric waves cannot propagate. Under the hypothesis of constant material coefficients, the propagation of several types of coupled first- and second-sound pulses, both in equilibrium and in non-equilibrium states, has been pointed out. Of particular interest are those waves called by the authors predominantly thermal and predominantly elastic.

In [5], the material model is developed within the frame of Extended Irreversible Thermodynamics [24]. The continuum system is rigid, the state space is local, as well as the evolution equation for *c*. The governing equation for the heat flux is of the Cattaneo type. It is proved that the speed of the heat pulses depends on *c* but does not depend on the gradient of *c*. The wave amplitude instead depends on such gradient and on the direction of propagation. For the sake of comparison, in Table 1, the results obtained in the present paper are compared with those obtained in [4,5].

The evolution of the wave amplitude has not yet been investigated for the model presented here, and it will be analyzed in a more general framework in a forthcoming paper. In fact, in future research, we aim at considering, for the same model, a more general set of restrictions with respect to Equations (42), in which we expect to offer a wider range of possibilities of nonlinear thermoelastic coupling. In particular, the state space can be enlarged by including the gradient of $E_{ij}$ so that the connection with the Mindlin strain gradient theory [46] can be analyzed.

## Figures and Tables

**Table 1:** Comparison of the results in [4,5] with the results obtained in the present paper (NI = Not Investigated; NE = Not Expected).

Results	Ref. [4]	Ref. [5]	Present Paper
Dependency of *s* and Tij on the gradient of the basic variables	No	NE	Yes
Dependency of the speed of propagation on *c*	NE	Yes	Yes
Dependency of the wave amplitude on c,i	NI	Yes	NI
Dependency on the direction of propagation of the wave amplitude	NI	Yes	NI
Propagation of pure stoichiometric waves	NE	No	Yes

## Data Availability

Not applicable.
